# Social Concern Theory and Family Violence Among Latino Families

**DOI:** 10.1177/08862605231218220

**Published:** 2023-12-11

**Authors:** Egbert Zavala, Krystlelynn Caraballo

**Affiliations:** 1 The University of Texas at El Paso, USA; 2 Arizona State University, Phoenix, USA

**Keywords:** Social Concern Theory, family violence, Latino families, familismo, code of the street

## Abstract

This paper examines whether Agnew’s Social Concern Theory can be applied to explain family violence perpetration among Latino families. Social concern theory maintains that people have biological inclinations to care for the welfare of others, desire close ties with certain individuals, follow certain moral intuitions, and conform to the behaviors of others. As such, this study tests whether an individual’s social capital (care about the welfare of others), familismo (desire for close ties), code of the streets (moral intuitions), and obligation to obey the police (conformity to social norms) is associated with family violence among a Latino sample. Using data from the *El Paso Neighborhood Survey Project*, which surveyed a random sample of 1,059 adults living in El Paso County, Texas in 2014, findings indicate that three of out of four theoretical constructs in the final model were found to be significant. Higher levels of social capital and familismo were associated with lower odds of perpetrating family violence, while code of the streets increased family violence. Obligation to obey the police was non-significant. Agnew’s theory modestly explained family violence among Latino families.

## Introduction

There has been a recent surge of research testing the core propositions of Agnew’s (2014) social concern theory (Choi et l., 2022; Chouhy et al., 2017; Craig, 2017; Hong et al., 2019; Kabiri et al., 2024; TenEyck & Barnes, 2019). Unlike traditional criminological theories that largely portray justice-involved individuals as being in the pursuit of self-interest (e.g., Gottfredson & Hirschi, 1990), social concern theory maintains that people have biological inclinations to care for the welfare of others, desire close ties with certain individuals, follow certain moral intuitions, and conform to the behaviors of others. These inclinations may lead some individuals to give more consideration to others than to themselves, reducing the probability of engaging in criminal behavior. Since its inception, however, no study has applied this theoretical framework to family violence, defined as “violence that can occur between any family members, such as violence between siblings or across generations, in addition to violence between intimate partners” (Curry et al., 2018, p. 171).

Exposure to or experiencing family violence has been associated with negative consequences such as intergenerational transmission of crime (Daquin et al., 2023), chronic mental illness, substance use, depression, and physical injury (Coker et al., 2002). Thus, it is pertinent for researchers and practitioners to unmask protective factors that may help reduce it. Further, scholars have called for more research on family violence among marginalized populations, including Latinos (Malley-Morrison & Hines, 2007). While studies have found Latinos experience family violence higher than, or comparable to, other racial and ethnic groups (Caetano et al., 2008; Cummings et al., 2013; Morrison et al., 2023), others have found family violence to be lower among Latinos (e.g., Curry et al., 2018; Wright & Benson, 2010). It is the latter for which social concern theory may provide a rationale for the phenomenon.

The purpose of this study is to provide an initial test of Agnew’s (2014) social concern theory, advancing the literature in at least three significant ways. First, we accept the invitation made by Zavala & Kurtz (2021) to explore and test other criminological theories that have yet to examine family violence. They argued that the theoretical frameworks used to explain this issue in the criminological literature are concentrated on social learning, general theory of crime, and general strain theory (e.g., Zavala & Krtz, 2021). Relying exclusively on these theories without testing others limits scholars from discovering new perspectives to help further understand—and prevent—family violence. Secondly, an important requirement for evaluating any theory is the exploration of core propositions with various populations and different types of behaviors. In this study, we use a Latino sample to capture a population not often centered in research (Brown, 2009) and focus on family violence, an outcome that has yet to be studied using this theoretical framework. Third, this study provides a more comprehensive test of the social concern theory. As we demonstrate in the literature review below, most research testing the core propositions of social control theory does not test all four elements in a single study.

These goals are accomplished through the following steps. First, we provide a synopsis of the family violence literature. Next, we provide an overview of the four major theoretical concepts of social concern theory, how each concept is pertinent to Latino culture, and studies that have tested this theory. From this literature review, we formed hypotheses to guide the empirical analysis. We then provide a detailed methodology section that explains how these data are uniquely suited for testing this theory. Finally, the results are presented and the paper concludes with a discussion of the findings, study limitations, and potential for future research.

### Family Violence Research

Family violence is defined here as “violence that can occur between any family members, such as violence between siblings or across generations, in addition to violence between intimate partners” (Curry et al., 2018, p. 171). Family violence incorporates violence including child abuse, intimate partner violence, and elder abuse (Malley-Morrison & Hines, 2004), each with their own sizable wealth of literature. Several studies have studied family violence among Latinos, with an emphasis on the cultural tenets that have acted as both protective and risk factors for family violence among Latinos: (a) familism (familismo); (b) machismo, marianismo, and respect; and (c) Catholicism, cultural fatalism, and folk beliefs (Curry et al., 2018; Malley-Morrison & Hines, 2004). Additionally, generational status and other measures of acculturation have been found to be significantly related to family violence (Curry et al., 2018). While each of these cultural tenets is important, we focus on those that we are able to test with the data: familismo and generational status. Familismo describes a strong sense of dedication and service to family (Zambrana, 2011) and fosters strong family ties and bonds.

### Agnew’s (2014) Social Concern Theory

Agnew’s (2014) social concern theory is composed of four theoretical constructs: care about the welfare of others, desire close ties to certain others, follow certain moral intuitions, and conform to the behaviors and views of others. The first is the notion that people will naturally care for the welfare of others. Agnew (2014) points to studies across disciplines demonstrating that people feel sad or distressed when innocent people suffer (empathy) and that people are willing to help those in need (sympathy), especially when they personally interact with these individuals. Therefore, Agnew (2014) reasoned that people are more likely to care about the welfare of others when they personally identify or have a strong feeling of “oneness” with those individuals. Hypothetically, people who show or indicate caring for others are less likely to engage in criminal behavior.

The second construct is a person’s desire to have close ties with certain individuals. Agnew (2014) states that people naturally want to establish close connections with certain individuals, desire to feel valued and accepted by others, and experience sadness or other negative emotions when they are rejected. The desire for close ties results in an inclination to cooperate with others, even at the expense of their own self-interest (Agnew, 2014).

The third element is an individual’s desire to follow certain moral intuitions. Again, Agnew (2014) notes that, generally, people agree with not killing or hurting innocent people, taking the property of innocent individuals by force or theft, or treating others in an unequitable manner (e.g., cheating or engaging in deceptive practices). Further, Agnew (2014) notes that people are also willing to punish anyone who violates these intuitions. Taken together, people with high moral intuitions are less likely to engage in criminal behavior because they would not want to hurt innocent people but are willing to punish others for violating social norms. While the current study is not able to test all moral intuitions suggested by Agnew (2014), it is able to examine the influence of one’s thoughts about the code of the streets.

Anderson (2000) states that the code of the street is a “cultural adaptation to a profound lack of faith in the police and judicial system—and in others who would champion one’s personal security” (p. 34). The two primary orientations of residents of inner-city neighborhoods are “street” and “decent.” Individuals who gravitate toward a street orientation “show a lack of consideration for other people and have a rather superficial sense of family and community” (p. 45). Such people are more likely to use violence to gain respect, prevent and resolve disputes, and encourage their children to defend themselves through violent encounters. Although decent people may also engage in violence for self-defense, these individuals are more likely to accept mainstream values and seek alternatives to violence to address disputes (Anderson, 2000). Thus, individuals who align more with the code of the street are likely to display lower levels of this moral intuition and, as a result, may be more likely to resort to family violence.

The last portion of the theory is a person’s inclination to conform to the behavior and views of others. Here, Agnew (2014) argues that people generally follow social norms and behave accordingly. Agnew (2014) also postulates that individuals who do not conform to these expected behaviors are more likely to be punished. Social concern is also affected by perceptions of others as “ingroups” or “outgroup” members. For example, Agnew suggests,

the “moralistic” crime motivated by social concern may sometimes be quite immoral in fact. This is most likely when ingroup members experience severe strain and attribute such strain to outgroup members who they believe have violated the inclinations for social . . . In such cases, outgroup members may be dehumanized and seen as a major threat. (p. 17)

Thus, it can be inferred that individuals who share a cultural or racial identity may be more likely to feel concern for others within their ingroup, but are also affected by subgroup identification and institutional factors.

### Latino Culture and the Impacts of Immigration

**Figure 1.**
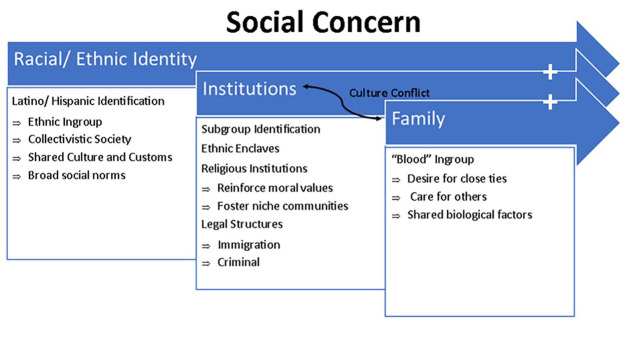
Layers of social concern among Latinos.

In order to contextualize the measures used to test the theoretical framework as well as provide a rationale for our hypotheses, a brief discussion of Latino culture is warranted. First, it is important to acknowledge that social concern is multifaceted and the expression of social concern may manifest in unique ways. Further, due to racial/ethnic discrimination, there may be a stronger sense of ethnic cohesion among Latino communities and families than among other groups (Chavez, 2013) (Figure 1).

Broadly, a shared ethnic identity provides a general “ingroup” by which individuals may identify with social values, economic interests, or cultural traditions. An interesting aspect of Agnew’s social concern theory is his discussion of “collectivistic” versus “individualistic” societies. Specifically, he states, “. . . evidence indicates that people in collectivistic societies . . . are higher in certain aspects of social concern, such as compassion and the need for social approval . . .” (p. 12). While not specifically discussed, Latino societies/cultures are often referred to as “collectivistic” due to a generally accepted emphasis on family and community cohesion (DiPietro & Cwick, 2014). Traditional Latino communities also commonly emphasize religiosity (primarily Catholicism), which is a key contribution to positive social networks and mechanisms of enforcing social and moral norms (Malley-Morrison & Hines, 2004). The collectivistic orientation of Latino culture is often attributed to greater social ties, economic opportunities, and informal social control (Lee & Martinez, 2002). Based on these factors, Latinos would be expected to demonstrate increased care for the welfare of others, desire for close ties with others, desire to follow certain moral institutions, and an inclination to conform to social norms. Theoretically, these propositions would lead us to expect that Latinos would be less likely to report perpetrating family violence.

Within the broader culture are structures that impact subgroups of the population such as laws, neighborhood dynamics, and local institutions. Within Latino communities, institutions are key enforcers of social norms, with religious institutions acting as a deterrent for spiritual reasons and law and immigration enforcement being a control mechanism to ensure compliance with American laws. However, these institutions may foster cultural conflict. For example, racial profiling and over-policing of minority communities have fostered distrust of police (Chavez, 2013), encouraging mechanisms of informal social control and an increased adherence to the code of the street. Further, some religious orientations may encourage traditional gender norms or condone violence between husbands and their wives in certain circumstances (Raj & Silverman, 2002). Likewise, the social norms of the country of origin may conflict with the host country’s laws or social norms (i.e., American), inciting friction within households. Prior research has found that culture conflict may increase incidents of family violence (Raj & Silverman, 2002; Reina et al., 2014) or pressure victims into silence (Stauffer, 2021).

There are key aspects of Latino culture that make this group particularly unique to study including the high proportion of migrant and mixed-status families. The intersection of Latino culture and immigration factors has the potential to produce null effects or disrupt the beneficial dynamics of social capital (Kubrin & Desmond, 2015; Stauffer, 2021). To illustrate, Kubrin and Desmond (2015) found little evidence to support that community social capital mediated the association between neighborhood immigrant concentration and adolescent violence. Further, as Stauffer (2021) finds, the benefits of social capital are constrained due to the emphasis on the collective instead of individual needs and structural factors as a result of undocumented or insecure legal status. Specifically, victims of gender-based violence reported “ambivalence” about disclosing violence to kin due to the potential for shame from within social networks or the increased risk of deportation of themselves or family members (Stauffer, 2021).

Finally, the family unit should, hypothetically, elicit the greatest social concern for others as family units typically have shared biological factors, care for others in their households, and elicit a desire for close ties (Agnew, 2014). As it pertains to the current study, Latino culture fosters a strong emphasis on familismo. This study would expect that respondents with a higher belief in familismo would be less likely to report perpetrating family violence, since abusing or otherwise hurting their partners or family members would cut the ties they strongly desire (Lackey & Williams, 1995). Therefore, it would be reasonable that Latinos who placed strong importance on familismo would be less likely to engage in family violence as suggested by social concern theory. However, it is important to note that in Stauffer (2021), Latino culture around familismo was a key reason why victims of gender-based violence placed their family before their own safety and remained silent about abuse. As the “family caretaker,” Latinas are especially vulnerable to pressure to maintain the family structure, even if requiring them to “bear quietly whatever pain may go with her role” (Madley-Morrison & Hines, 2004).

### Prior Studies

Since its inception, a growing body of studies have tested social concern theory’s core components, but most of these studies are partial tests of select components (Craig, 2017; TenEyck & Barnes, 2019) or populations (Hong et al., 2019). For example, Craig (2017) tested the theory’s efficacy on white-collar and street crime, finding that low empathy (the study’s measure for caring for the welfare of others) is significant in predicting credit card fraud, embezzlement, and shoplifting. In addition, a person’s affective empathy, or their ability to experience feelings of compassion and warmth for others, predicted shoplifting intentions, credit card fraud, and embezzlement, while cognitive empathy (or a person’s ability to take the views of others) was found to be protective against shoplifting and credit card fraud. This study also found that low self-control mediated the relationship between empathy and intentions to shoplift and embezzle. However, this study did not test other concepts from the theory.

TenEyck and Barnes (2019) provided another partial test when they applied social control theory to criminal behavior using data collected from the *National Longitudinal Study of Adolescents*. Their study showed that their measure of caring for others was negative and significant in predicting criminal behavior as adults as suggested by the theory. Perhaps more importantly, their study showed that one of the reasons why females commit less crime than males is because they tend to care more for others than males. Again, this study is limited because it did not contain measures for close ties, moral intuitions, or conforming to others. Hong et al. (2019) provide another partial test of the theory to understand the delinquent behavior of Korean adolescents. This study showed that strong teacher bonding strengthens a youth’s social concern (a combination of the inclination to follow moral intuitions and caring for the welfare of others), which in return was found to be negative and significant in predicting delinquency. While informative, this study did not have measures for close ties with others, nor measures for the tendency to conform to others and to social norms.

Chouhy et al. (2017) tested the theory on delinquency using data collected from the *Pathways to Desistance* project. Unlike the previous studies, this research examined all four components of social concern theory. This study found that only two out of the four concepts predicted delinquency. Specifically, the authors found that juveniles who lacked empathy were more likely to report engaging in delinquency, while juveniles with higher levels of consideration for others reported less delinquency (the study’s measures for caring for the welfare of others). Moral intuitions (measured by a juvenile’s moral disengagement) was found to be negative and significant with delinquency. The remaining two constructs of social concern theory were not found to be significant in their study (e.g., desire for close ties and desire to conform to others). Social concern mediated some measures obtained from social learning, strain, social bonding, self-control, and social support with delinquent behavior.

## The Current Study

A small but growing body of literature has begun testing the core propositions of Agnew’s (2014) social concern theory. While these studies are informative, no study to date has determined whether social control theory is applicable to family violence among Latinos. There is reason to believe that social concern theory would be appropriate to explain lower levels of family violence among Latino families given that culturally specific characteristics align with the theory’s theoretical concepts, but have yet to be empirically examined. Based on this gap, and grounded on previous research, this paper examines the following hypotheses:

**Hypothesis 1:** Social capital will be significantly and negatively associated with perpetrating family violence among Latinos.
**Hypothesis 2:** Familismo will be significantly and negatively associated with perpetrating family violence among Latinos.
**Hypothesis 3:** Adherence to the code of the streets will be significantly and positively associated with perpetrating family violence among Latinos.
**Hypothesis 4:** Obligation to obey police will be significantly and negatively associated with perpetrating family violence among Latinos.

## Methods

### Data

The data used herein are drawn from the *El Paso Neighborhood Survey Project*, which surveyed a random sample of 1,059 adults living in El Paso County, Texas in 2014 (Curry et al., 2017). As described in Curry et al. (2018), potential respondents were contacted first by mail and then in person by trained interviewers who scheduled face-to-face interviews or conducted them on the spot if participants, who were paid $20, preferred. Face-to-face interviews were conducted in either Spanish or English, depending on the preference of each respondent.

The analysis here uses—due to the hypothesized relationships among Latino individuals, a subsample of 863 respondents (81.5%) who identified as Latinos. Of these, 583 (67.56%) were born in the United States, 269 (25.4%) were born in Mexico, and 13 (1.51%) were born elsewhere. This high percentage of Mexican-born foreign nationals is not surprising due to El Paso’s location along the U.S.–Mexico border in far west Texas, a traditional migrant destination. However, survey items seeking to ascertain documentation status resulted in a high percentage of missing cases, making analyses unfeasible with these data. After listwise deletion accounting for the control variables, the final Latino subsample was 848 respondents. Survey items measuring income also received a high percentage of non-responses, so this variable is not included in analyses. Further design and methodological information is available elsewhere (Curry et al., 2018) and on the Inter-university Consortium for Political and Social Research website (https://www.icpsr.umich.edu/web/ICPSR/studies/38247).

### Dependent Variable

Questions used to capture family violence were based on the revised *Conflict Tactics Scale* (CTS2; Straus et al., 1996), but expanded to include sexual violence and verbal abuse (Curry et al., 2018). This scale focused on the household level rather than the individual or intimate partner dyad. Specifically, respondents answered a series of 10 yes-or-no questions asking if they are aware of any household member (including themselves) having engaged in any acts of family violence toward another household member (including themselves) in an effort to hurt that person. Example questions include: “A household member threw something that hurt another household member,” “A household member twisted the arm or hair of another household member,” and “A household member kicked, punched, or hit another household member, or hit a household member with a weapon or object.” Preliminary analyses showed that this measure was highly skewed with about 81% of respondents indicating that no instances of family violence occurred during the study period. To address this distribution problem, subsequent analyses use a binary measure of family violence whereby zero (0) indicates no incidences of family violence and one (1) indicates that at least one incidence of family violence occurred.

### Independent Variables

#### Care About the Welfare of Others

Six questions were developed and used by Curry et al. (2017) to gauge social capital. Respondents were asked if the people in their neighborhood would “. . . help each other find a job (or a better job),” “. . . help each other improve their job skills,” “. . . help each other find a good place to live,” “. . . help each other find good places for their children to play or have fun,” “. . . help each other deal with governmental officers or agents and governmental procedures,” and “. . . help each other obtain or complete governmental documents, such as those regarding employment, taxes, and establishing legal citizenship.” Respondents recorded their responses using a 5-point Likert-type scale (1 = *Strongly Disagree* to 5 = *Strongly Agree*). These six questions were summed into an index, with higher scores indicating higher levels of social capital (α = .90).

#### Desire for Close Ties

*Familismo* was used to capture desire for close ties and measured using the subset of *The Latino/a Values Scale* developed by Kim et al. (2009). A total of five questions captured familismo: “A mother must keep the family unified,” “One’s family is the main source of one’s identity,” “One should never bring shame upon one’s family,” “One’s family is the main source of support,” and “The needs of the family are more important than my own individual needs.” Respondents recorded their responses using a 5-point Likert-type scale (1 = *Strongly Disagree* to 5 = *Strongly Agree*). These five questions were summed into an index, with higher scores indicating higher levels of familismo (α = .78).

#### Moral Intuitions

*Code of the Streets* was used to capture the concept of moral intuitions based on Stewart and Simons’ (2010) scale. Specifically, respondents were asked, “Teenagers and young adults in your neighborhood must be willing to fight to gain respect among their peers,” “Parents in your neighborhood teach their kids to fight back if they are insulted or threatened,” “People in your neighborhood will seek revenge even if it means resorting to violence if a loved one is disrespected,” “Young men in your neighborhood try to act tough,” “Young women in your neighborhood try to act tough,” “Young men . . . who own guns or other weapons are often looked up to and respected by others,” “Young women . . . who own guns or other weapons are often looked up to and respected by others,” “People in your neighborhood do not respect a young man who is afraid to fight physically,” “People in your neighborhood do not respect a young woman who is afraid to fight physically,” “In your neighborhood, it is important to show others that a person cannot be intimidated,” and “People in your neighborhood would not help the police if they are investigating a crime.” Participants recorded their responses using a 5-point Likert-type scale (1 = *Strongly Disagree* to 5 = *Strongly Agree*). These 11 items were summed into an index, with higher scores meaning more adherence to the code of the streets (α = .90).

#### Conformity to Social Norms

*Obligation to Obey the Police* was used to capture conformity to social norms and measured with the following three questions as developed by Jackson et al. (2012): “You should do what the police tell you, even if you disagree,” “You should accept decisions made by the police, even if you think they are wrong,” and “You should do what the police tell you to do, even when you don’t like the way they treat you.” Respondents recorded their responses using a 5-point Likert-type scale (1 = *Strongly Disagree* to 5 = *Strongly Agree*). These three questions were summed into an index, with higher scores meaning a greater sense of obligation to obey the police (α = .85).

### Demographic and Control Variables

Prior research has demonstrated that sex (Johnson, 2008), age (Spencer et al., 2019), and 1.5 generational status (Curry et al., 2018) are correlated with family violence and, therefore, these variables are controlled for in this study. *Sex* is coded 1 for males and 0 for females. *Age* is measured in years. The 1.5 generation is often defined as foreign-born individuals who migrated at a young age and spent their formative years in the United States (DiPietro & Cwick, 2014). In this study, the 1.5 generational status was determined by asking where the respondent and their mother were born. If the respondent and their mother were born in Mexico, but moved to the United States before age 15, then the respondent was coded as a 1.5 generational migrant (see Curry et al., 2018).

#### Analytical Plan

The analyses are conducted in two steps using Stata 16. First, sample characteristics are presented, providing an overview of the distribution of the measures used. Second, given the binary nature of the dependent variable, logistic regression models are produced to determine which predictors are correlated with the dependent variable (Weisburd & Britt, 2014). To gauge if multicollinearity is a problem in these data, tolerance and variance inflation factors (VIFs) were calculated. All tolerances are above 0.20 and all VIFs are below 4, indicating that multicollinearity is not a problem in this study (Walker & Maddan, 2020; Weisburd & Britt, 2014). To account for the multistage cluster sampling design of the data, the Stata options “robust” and “cluster” were used to produce the standard errors.

## Results

Table 1 summarizes the descriptive statistics for all the study’s variables. Looking at the dependent variable, 81% of respondents did not report experiencing family violence. Moving to the main independent variables, social capital has an average of 20.5 on a range from 6 to 30, while familismo has an average of 21.5 on a range from 10 to 25. The code of the streets has a mean of 23.7 on a range from 10 to 48, while obligation to obey the police has a mean of 9.9 on a range from 5 to 15. Turning our attention to the control and demographic variables, the majority of respondents are female (57.7%) and the average age is 42 years old. Based on their responses to country of birth questions, around 10% of respondents are coded as 1.5 generation migrants.

**Table 1.** Descriptive Statistics (*n* = 848).

Variable	Coded	*N*	Mean (%)	*SD*	Range	VIF/Tolerance
**Dependent variable**						
Family violence perpetration	0 = No	687	(81.0)			
	1 = Yes	161	(19.0)			
**Independent variables**						
*Care about welfare of others*						
Social capital	6-Items	848	20.5	4.80	6/30	1.06/0.94
*Desire for close ties*						
Familismo	5-Items	848	21.5	3.15	10/25	1.04/0.95
*Moral intuitions*						
Code of the streets	11-Items	848	23.7	7.54	10/48	1.02/0.97
*Conformity to social norms*						
Obligation to obey police	3-Items	848	9.9	3.07	3/15	1.03/0.97
**Demographic and control variables**						
Sex	0 = Female,	489	(57.7)			1.03/0.97
	1 = Male	359	(42.3)			
Age	In years	848	41.1	16.34	18/85	1.03/0.97
1.5 Generation	0 = No	764	(90.1)			1.00/0.99
	1 = Yes	84	(9.9)			

*Note. SD* = standard deviation; VIF = variance inflation factor.

Table 2 displays the results of the logistic regression analysis. This table indicates that all but one of the social concern theory constructs—conformity—are significantly associated with family violence. Social capital is negative and significant. Higher levels of social capital decreased the odds of family violence perpetration by 5% (odds ratio [OR]  = 0.95). Furthermore, familismo is also negative and significant. Higher levels of familismo decreased the odds of family violence perpetration by 8% (OR = 0.92). Alternatively, code of the streets is positive and significant. Adherence to the code of the streets increased the odds of family violence perpetration by 6% (OR = 1.06). Finally, obligation to obey the police is not significant in these data. Finally, only two of three control and demographic variables were significant in this model. Age is negative and significant, meaning that being older decreased the odds of family violence perpetration by 3% (OR = 0.97). Respondents reporting being a 1.5 generation migrant increased the odds of family violence perpetration by 105% (OR = 2.05). Sex was not significant in these data.

**Table 2.** Logistic Regression Analyses Predicting Family Violence (*n* = 848).

	Model 1		
Variables	*OR*	*SE*	[95% CI]
**Demographic and control variables**			
Sex	1.02	0.22	[0.70, 1.47]
Age	0.97**	0.01	[0.96, 0.98]
1.5 Generation	2.05**	0.52	[1.19, 3.52]
**Independent variables**			
*Care about welfare of others*			
Social Capital	0.95*	0.02	[0.92, 0.99]
*Desire for close ties*			
Familismo	0.92**	0.03	[0.87, 0.98]
*Moral intuitions*			
Code of the streets	1.06**	0.01	[1.04, 1.09]
*Conformity to social norms*			
Obligation to obey police	1.04	0.03	[0.98, 1.11]
Constant	0.78	0.63	[0.16, 3.67]
χ=	59.26		
-2 Log Likelihood=	−375.68		
Pseudo *R*^2^=	0.09		

*Note. SE* = standard error; CI = confidence intervals; OR = odds ratio.

* *p* ≤ .05. **p ≤ .01.

## Discussion

Agnew’s (2014) social concern theory challenges the notion that people commit crimes in order to benefit themselves or their self-interest. Such an assumption, he argues, ignores a person’s natural inclination to care for the welfare of others, their desire to have close ties with people, follow certain moral intuitions, and conform to the views of others and social norms. The greater degree to which someone possesses these traits, the less likely they are to engage in crime. Since its foundation, a small but growing number of studies have tested its core assumptions. However, no study to date has examined its applicability to family violence among the Latino population. Data obtained from the *El Paso Neighborhood Survey Project* were used to determine the efficacy of social concern theory on family violence. Hypotheses were tested using a multivariate regression model and the analyses revealed several interesting findings.

Hypothesis 1 stated that social capital will be significantly and negatively associated with perpetrating family violence among Latinos. This hypothesis was derived from Agnew’s (2014) contention that individuals would be less likely to engage in criminal behavior when they cared about the welfare of other people. Our results support this hypothesis. The data demonstrated that Latinos who reported high levels of social capital were less likely to engage in family violence. This result is consistent with prior studies that have tested this theory (Chouhy et al., 2017). One possible explanation from this finding is provided by a study that found that greater access to social capital decreased acculturative stress, depression, and anxiety (Valencia-Garcia et al., 2012), three risk markers found to be correlated with family violence (Caetano et al., 2007; Spencer et al., 2019). While the current data does not contain measures for acculturative stress, depression, or anxiety, we encourage future research to test this possibility to determine if this is indeed what is happening.

Hypothesis 2 declared that familismo will be significantly and negatively associated with perpetrating family violence among Latinos. This hypothesis was established to test Agnew’s (2014) notion that people naturally want to establish close connections with certain individuals as well as their desire to feel valued and be accepted by others. One cultural feature of Latino families is the concept of familismo, which describes a strong sense of dedication and service to family and, thus, fosters strong family ties and bonds. It is reasonable to assume that respondents with a strong sense of familismo would be less likely to perpetrate family violence. As evident in the data, this hypothesis is supported. Individuals with high levels of familismo were less likely to perpetrate family violence. This result is consistent with other studies that have reported a relationship between familismo and dating violence among teens and emerging adults (East et al., 2010; Folger & Wright, 2013). As noted earlier, Latinos who placed strong importance on familismo may be less likely to engage in family violence because hurting their partners or family members would compromise familial bonds. Our results reinforce familismo as an important protective factor.

Hypothesis 3 claimed that adherence to the code of the streets would be significantly and positively associated with perpetrating family violence among Latinos. Our findings support that adherence to the code is positively associated with perpetrating family violence. This is consistent with prior studies (Chouhy et al., 2017). Recall that Agnew (2014) argued that those with strong moral intuitions are less likely to engage in crime. Based on Anderson’s (2000) conceptualization of the code of the street, stronger adherence to the code would align with lower moral intuitions, as violence is a primary means of obtaining respect. Thus, it is reasonable to conclude that families (or family heads) who adhere to the code are more likely to use violence to demand respect or compliance from other relatives. Given these findings, intervention and prevention programs should target code of the streets beliefs in families to reduce family violence (Banse et al., 2013).

Hypothesis 4 expected that obligation to obey police would be significantly and negatively associated with perpetrating family violence among Latinos. This hypothesis is not supported. Obligation to obey police was positive, but not significant. This counterintuitive finding may have multiple potential explanations. First, it is possible that our operationalization of Agnew’s concept—conformity to social norms—is too narrow. Additionally, it is possible that statistical power is a factor and that the sample lacks sufficient cases to adequately disentangle this relationship. Alternatively, it is possible that culture conflict plays a role. Prior research has found that IPV/family violence is “maintained by societies because of culture, social context, and laws that often uphold male control of female partners” (Raj & Silverman, 2002, p. 369). In their systematic review of violence against immigrant women, Raj and Silverman (2002) found that culturally traditional gender roles may facilitate violence. For example, both men and women reported that, in some cultures, it is acceptable for men to discipline women using physical force (Raj & Silverman, 2002). Among migrants, the culture conflict between the country of origin and the United States may exacerbate family violence while not necessarily being identified as wrong. As Raj and Silverman (2002) note, “Many immigrants, including batterers, are not aware or accepting of IPV as a criminal offense” (p. 370). Further, family violence may be deemed a private matter and victims may feel shame or be unwilling to report abuse (Fuchsel, 2013; Reina et al., 2014). Finally, the very notion of familismo discussed earlier may lead victims to prioritize the family unit (including the perpetrator) over their own well-being (Reina et al., 2014; Stauffer, 2021). Further research is needed to delineate these effects.

Several demographic and control variables were found to be significant and, therefore, warrant further discussion. First, contrary to Agnew’s hypothesis that women would demonstrate more social concern, our data did not reveal a significant difference between males and females. Next, older respondents were less likely to report acts of violence than younger respondents. This finding may not be surprising given that older respondents are less likely to form intimate relationships where the occurrence of violence increases. In addition, younger individuals may be in more relationships or just started dating whereby acts of violence are more likely to occur.

An interesting finding is the significantly elevated odds of the 1.5 generation engaging in family violence. A longstanding body of literature finds that acculturation to American society increases conflict between first generation parents and children born or raised in the United States (DiPietro & Cwick, 2014), increasing delinquency (Bersani et al., 2014). However, the 1.5 generation often demonstrates lower rates of delinquency and violence than later generations. DiPietro and Cwick (2014) argued that adherence to familism is more nurtured in females than males, thus there may also be an interactive effect between generational status and sex. Although this study does not test this interaction, it may provide fruitful considerations for future work.

While it would be premature to make policy recommendations on a single study, we hope that these findings will contribute to the broader collection of work that aims to inform family violence prevention programs. Addressing some of these via policy may be problematic as it may target aspects of Latino culture resulting in further stereotyping and discrimination. Scholarship has long recognized that aspects of Latino culture may act as both protective and risk factors for family violence (Curry et al., 2018; Malley-Morrison & Hines, 2007). Agnew’s social concern theory highlights the positive influence that aspects of Latino culture can have on reducing family violence. With further research, this framework may help explain the rates of family violence across groups and improve programs aimed at reducing violence.

### Limitations

The results of this study should be viewed with several limitations in mind. First, the cross-sectional nature of the data does not allow for a clear determination of the causal relationship between the study variables and perpetration of family violence. In other words, the temporal ordering of events is unclear. Therefore, there should be an effort to collect longitudinal data with these variables of interest in mind. Secondly, some respondents may have been reluctant to disclose their perpetration due to social desirability. Underreporting has been particularly problematic among Latinos and migrants (Gutierrez & Kirk, 2017). Third, data limitations prevented us from incorporating other variables found to influence family violence such as sexual promiscuity and exposure to violence in the community (Gover, 2004; O’Keefe & Treister, 1998). Additionally, institutional influences such as religion and immigration law cannot be measured using the present data. Such exclusion may have altered our results. Notably, religiosity and immigration factors may increase conformity to social norms or introduce cult conflict that disrupts the positive impact of social concern. Fourth, the conflict tactics scale has been criticized for not capturing the context in which violence occurs or the motivations for using violence (Kimmel, 2002). Finally, although the sample was large, it is not a nationally representative sample of Latino families. The nationality/ancestry of U.S.-born Latinos was not collected and thus, we are unable to disaggregate “Latino” into the numerous cultures that comprise this panethnic label. Scholars have noted the need to disentangle these heterogeneous groups in research (Malley-Morrison & Hines, 2007). Additional research on Latinos is necessary to delineate the impact of social concern on family violence and other outcomes. Thus, caution should be exercised when generalizing these results to other Latinos living outside El Paso County, Texas.

## Conclusion

The primary purpose of this study was to provide an initial test of Agnew’s (2014) social concern theory as a potential theoretical framework to study family violence among Latinos. This study advanced prior work by (a) testing all four components of social control theory instead of providing a partial test, (b) emphasizing a Latino sample, and (c) applying this theoretical framework to family violence, an outcome that has not previously been explored in this context. This study demonstrated that elements of social concern theory were significantly related with family violence and that results may vary across subgroups. Thus, social control theory should be considered a potential explanatory framework for understanding family violence. While this study was unable to make causal claims, we recommended that future researchers replicate this study using nationally representative data in the hopes of creating more prevention and treatment programs grounded on social concern theory to address family violence.
